# A Comprehensive Review of SURGICEL®: Efficacy, Applications, and Radiological Insights in Surgery

**DOI:** 10.7759/cureus.103048

**Published:** 2026-02-05

**Authors:** Dia R Halalmeh, HusamEddin Z Salama, Yusuf-Zain Ansari, Carmelo V Venero, Saad A Farooqui, Marc D Moisi

**Affiliations:** 1 Department of Neurosurgery, University of Iowa Hospitals and Clinics, Iowa City, USA; 2 Department of Neurosurgery, Hurley Medical Center, Flint, USA; 3 Department of Biology, Temple University, Philadelphia, USA; 4 Department of Neurosurgery, University of Connecticut Health, Farmington, USA; 5 Department of Neuroscience, Temple University, Philadelphia, USA

**Keywords:** clinical applications, complications of hemostatic agents, oxidized regenerated cellulose (orc), radiological considerations, surgical hemostasis, surgicel®, topical hemostatic agents

## Abstract

SURGICEL® is a widely used hemostatic material designed to control capillary, venous, and small arterial bleeding. Introduced in 1960, it functions by producing cellulosic acid, which promotes platelet aggregation and activation of the coagulation pathway. This bioabsorbable material has been utilized in various surgical procedures to manage perioperative bleeding. The objective of this article is to review the mechanism of action, clinical applications, and imaging characteristics of SURGICEL®, as well as its role in differential diagnosis in surgical contexts. This article serves as a comprehensive review of SURGICEL® in various surgical fields, including neurosurgery, urology, orthopedics, and maxillofacial surgery, while also examining SURGICEL®’s hemostatic mechanism, clinical efficacy, and bioabsorbable properties. The review also evaluates its radiological appearance in ultrasound, CT, and MRI, and addresses its role in differential diagnosis and potential complications, such as granuloma formation and infections, based on existing literature. SURGICEL® has proven effective in achieving hemostasis in a wide range of surgical procedures, including maxillofacial and neurosurgical operations. As a bioabsorbable material, it induces minimal tissue reactions and is gradually absorbed over time. On imaging, it appears as echogenic masses, radiopaque bands, or alterations in signal intensity, particularly on MRI. MRI can differentiate SURGICEL® from adjacent tissues, aiding in the identification of hematoma-like masses or complications such as granulomas and infections. Overall, SURGICEL® offers significant utility in surgical hemostasis with minimal side effects. However, careful monitoring is required to avoid complications, and knowledge of its radiological appearance is essential for accurate differential diagnosis. This review highlights its coagulation-promoting properties, imaging features, and potential complications to guide its effective clinical use.

## Introduction and background

Effective hemostasis is of utmost importance for achieving optimal surgical outcomes. First, it helps minimize blood loss, which is essential for maintaining hemodynamic stability in patients and reducing the need for blood transfusions, thereby decreasing the risk of associated complications. Additionally, effective hemostasis supports faster surgical times, reducing overall procedure duration and the associated risks of anesthesia and postoperative complications. Furthermore, it promotes better wound healing by reducing the formation of hematomas and seromas, which can lead to infections and delayed recovery [1,2].

Topical hemostatic agents play a crucial role in modern surgical management, especially in the management of perioperative bleeding. Intraoperative bleeding remains one of the most common perioperative complications and significantly affects the morbidity and mortality of patients in the perioperative period, particularly with the aging population, which is associated with an increased prevalence of comorbidities and greater use of anticoagulants [1,3,4]. Choosing the appropriate hemostatic agent depends on several factors, including the type and severity of bleeding, the anatomical site, patient characteristics, coagulation and comorbidity status, and the surgeon’s preference and expertise. In clinical practice, compression with cotton gauze and wound closure with sutures or staples are the most common methods to stop bleeding. While traditional hemostatic techniques such as suturing, electrocautery, and vessel ligation play a pivotal role in achieving hemostasis, there are scenarios where these methods prove insufficient. In such cases, topical hemostatic agents emerge as valuable adjuncts to control bleeding effectively [5].

Various types of hemostatic agents are employed in surgical settings, categorized as physical agents and biologically active agents. These agents differ in physicochemical properties and mechanism of action, as well as in ease of use, safety, and cost. Physical hemostatic agents function by providing a scaffold or substrate for clot formation, and they are directly applied to bleeding surfaces during surgery. Examples of physical agents include absorbable gelatin sponges, oxidized cellulose, and collagen-based products. Biologically active hemostatic agents, on the other hand, work through various mechanisms such as promoting platelet aggregation, activating clotting factors, or enhancing fibrin formation. These agents may include thrombin, fibrin sealants, and synthetic hemostatic peptides [5].

SURGICEL® is a sterile, fabric or sponge hemostatic bioabsorbable material composed of oxidized regenerated cellulose polymer (multimer of polyanhydroglucuronic acid). It was manufactured by the Ethicon subsidiary of Johnson & Johnson and introduced into clinical practice in 1960 mainly to assist in the control of post-surgical bleeding, providing the unique feature of superior handling characteristics compared to traditional surgical sponges and less conformable agents. Unlike its predecessors, SURGICEL® was distinguished by its plant-based origin, avoiding the risks associated with human or animal-derived products [6-8]. Similar to other types of physical hemostatic agents, oxidized regenerated cellulose (ORC), including SURGICEL®, works by promoting hemostasis through several mechanisms. It acts as a physical barrier and can lead to platelet aggregation and contact activation of the intrinsic coagulation pathway. Additionally, SURGICEL® has a pH of 3; the application of this acidic polymer creates an acidic environment that leads to lysis of red blood cells, accounting for the brownish discoloration after application. This further accelerates clotting and acts as a scaffold to facilitate platelet adhesion and clot formation, as well as promotes vasoconstriction [5-7]. This multifaceted mechanism, alongside its chemical makeup and low pH, is just one of the factors contributing to its effectiveness as a hemostatic agent. Furthermore, ORC absorbs fluids from the surrounding tissues, concentrating clotting factors and promoting the formation of a stable blood clot [9]. This innovation offered a new direction in the development of hemostatic agents, emphasizing biocompatibility and safety.

ORC has been shown to have an effect on bacterial growth. ORC products, such as SURGICEL®, create a physical barrier at the site of application, which can help reduce the risk of bacterial contamination and infection. Additionally, ORC has been found to have some antimicrobial properties, although the exact mechanisms are not fully understood. Studies have suggested that ORC produces coagulative necrosis with a tamponade effect and has been demonstrated to have in vitro bactericidal activity as well as antibacterial activity in an animal model of wound healing. It may help inhibit the growth of certain bacteria, including *Staphylococcus aureus* and *Escherichia coli*, which are common causes of surgical site infections [10,11].

**Figure 1:**
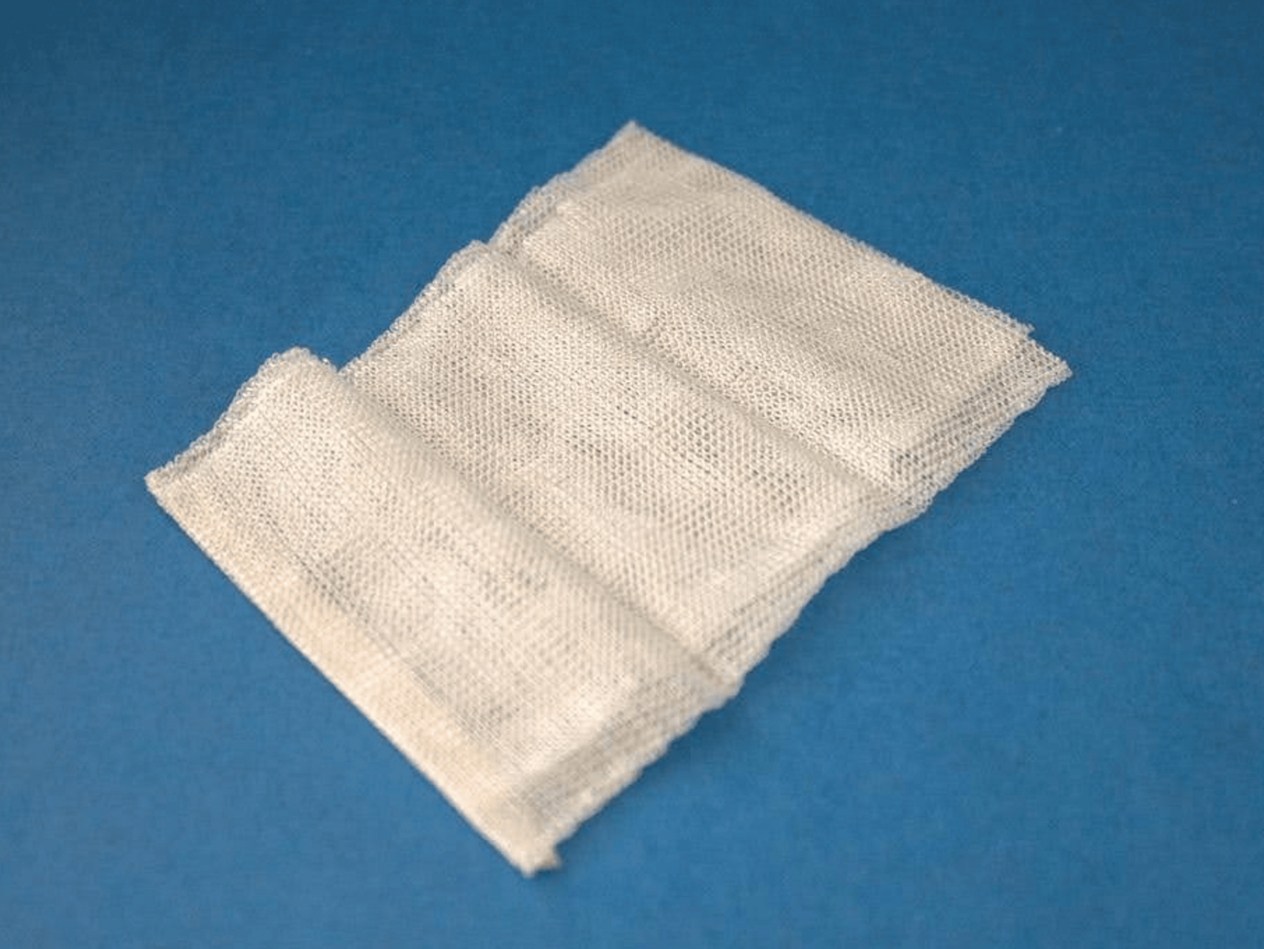
SURGICEL® gauze. This image displays a sample of SURGICEL® gauze. SURGICEL® gauze is a sterile, bioabsorbable hemostatic fabric used in surgical procedures to control capillary, venous, and small arterial bleeding. Courtesy of Kristin N MacPhee.

SURGICEL® is utilized to control capillary, venous, or smaller arterial bleeding in various open, laparoscopic, and endoscopic surgical procedures, including post-surgical bleeding and superficial injuries to the skin [1,5,12]. It is available in various forms, including mesh, gauze (Figure 1), fibrillar tufts, sponges, sheets, powder, and woven fabrics, making it suitable for use in a wide range of surgical procedures and anatomical locations. It is biodegradable and gradually absorbed by the body over time, eliminating the need for removal in most cases. ORC is generally well-tolerated, with low risk of adverse reactions or tissue damage. However, as with any hemostatic agent, the use of ORC should be tailored to the specific clinical scenario, taking into account factors such as the type and severity of bleeding, anatomical considerations, and the patient’s overall health status.

Accurate identification of SURGICEL® on postoperative imaging is clinically vital. Misinterpreting these bioabsorbable materials as abscesses or tumor recurrences can lead to significant patient morbidity, including unnecessary invasive biopsies, prolonged hospitalizations, and avoidable secondary surgical interventions.

This narrative review aims to discuss the structural features of SURGICEL® and consolidate historical developments with current knowledge. This article also aims to identify persisting challenges and recent outcomes to inform future research and innovation on topical hemostatic materials. To fully appreciate the modern role of SURGICEL®, it is first necessary to examine its historical development alongside other foundational hemostatic agents.

## Review

Historical evolution

The development of the four major classes of topical hemostatic agents, namely, fibrin sealants, gelatin-based products, oxidized cellulose, and collagen products, has significantly evolved over the past century. While gelatin-based products, oxidized cellulose, and collagen products have remained relatively static since their initial inception, fibrin sealants have experienced a gradual, yet promising development [13]. First introduced in 1909 by Bergel, fibrin sealants are credited with being the first topical hemostatic agent in the modern surgical era [14]. The initial excitement around early fibrin sealants was tempered by the risk of infectious transmission, as these early formulations were derived from human plasma components. Although fibrin sealants were one of the first modern hemostatic agents, the issues experienced with them highlighted the need for safer hemostatic alternatives, leading to the exploration of other materials. Gelatin-based products such as Gelfoam, introduced in the 1940s, have undergone very little evolution since their first development. In fact, Gelfoam and Surgifoam are still offered in the same way as they were offered in their initial release [13]. The latest development in hemostatic agents has been collagen-based products. Introduced in the early 1970s, collagen-based products such as Avitene have become staples in surgical settings. These agents have remained relatively unchanged since their inception, focusing on minor modifications to their format and preparation rather than significant evolution. The brand Avitene has remained in a topical powder form since its release [13].

ORC is a physical hemostatic agent commonly used in surgery to control bleeding. It was first used in 1940, and later brought to the clinical market as SURGICEL® in 1960 by Johnson & Johnson [6,13]. It is derived from natural cellulose, typically sourced from cotton, which undergoes a process of controlled oxidation and regeneration by nitrogen dioxide to create a biocompatible and absorbable material. The oxidized regenerated form then serves as a platform for platelet aggregation and contact activation of the intrinsic coagulation pathway. It can be left in the surgical bed without significant local tissue reactions [13].

Surgical outcomes, clinical applications, and characteristics

SURGICEL® is commonly used in a variety of surgeries for its hemostatic properties, which help control bleeding during and after surgical procedures, particularly in cases where conventional methods of hemostasis, such as sutures or cautery, are ineffective or impractical. One of the key advantages of SURGICEL® is its ease of use. It can be easily cut to size and applied directly to the bleeding site, where it conforms to the shape of the tissue and helps seal the wound. This is also true because SURGICEL® is also available in a variety of shapes and structures. For example, SURGICEL™ Original, SURGICEL® SNoW™, SURGICEL® Powder, SURGICEL® Fibrillar®, and SURGICEL® Nu-Knit. Each of these forms of SURGICEL® has different physical characteristics that make it more suitable for different forms of surgery. For example, SURGICEL® Powder was designed to aid in mild-to-moderate intensity bleeding in a range of tissue surfaces, including broad or raw surfaces, and in locations where access or placement of fabric ORC products may be challenging or impractical [1]. Another example, SURGICEL® SNoW Absorbable Hemostat can be cut to size for use in endoscopic procedures [1].

Furthermore, SURGICEL® knitted strip (SURGICEL® Original) is readily conformable into a variety of shapes when cut into small pieces. However, it can be easily displaced during suctioning. A cottonoid patty is therefore placed on the SURGICEL® strip to allow for careful positioning using the tip of the suction device while achieving adequate hemostasis [7]. Conversely, the fibrillar form of SURGICEL® (SURGICEL® Fibrillar) allows direct suctioning without the need for cottonoid patty. SURGICEL® fibrillar is composed of a layered material that conforms to irregular surfaces and to regions that are not easily accessible. In addition, it remains moldable for longer periods of time compared to other forms, allowing the surgeon to reshape it within the surgical field. Furthermore, if bipolar cautery is needed, it can be applied directly through the saline-soaked layers. Encountered oozing during the surgery can be managed with these forms of SURGICEL®. Once adequate hemostasis is achieved, the SURGICEL® mass with the absorbed bleeding should be removed to avoid obscuring the surgical view, particularly in microneurosurgical intraspinal surgeries with deep and narrow surgical corridors [7]. SURGICEL® Nu-Knit is a heavily woven design of the original SURGICEL® and is useful during heavy bleeding. Non-regenerated oxidized cellulose is a recent, more potent form of SURGICEL® due to its unorganized fiber structure and larger density of hemostatic material. This slightly alters its appearance on postoperative imaging studies in comparison to other forms of SURGICEL®.

SURGICEL® is also absorbable, meaning that it is gradually broken down and absorbed by the body over time, eliminating the need for a second surgery to remove it. In this section, we will focus on the role of SURGICEL® in different surgical procedures and how it helps in maintaining hemostasis during surgery and thus improving surgical outcomes. These diverse structural forms allow for specialized applications across a wide array of surgical disciplines, beginning with complex abdominopelvic procedures.

Abdominopelvic Surgeries

SURGICEL® is a safe and effective adjunctive tool for controlling mild-to-moderate bleeding during abdominal surgeries ranging from obstetrics and gynecology, colorectal, urological, and others. It is particularly useful in surgeries involving highly vascular organs, such as the liver, spleen, or kidneys, where bleeding can be difficult to control [1,15]. It also helps in tissue dissection and identification of supplying blood vessels, and thus controls blood vessels locally. SURGICEL® finds utility in various abdominal procedures. In this section, we will discuss some of SURGICEL®’s uses in different abdominopelvic surgeries.

Indications and comparative use in hepatobiliary surgery: Similar to other abdominal surgeries, intraoperative bleeding remains one of the most common complications after hepatobiliary operations, especially in certain operations. For example, several studies suggested that the incidence of uncontrolled intraoperative bleeding was higher among patients with acute cholecystitis when compared to other subtypes of cholecystitis in patients undergoing early laparoscopic cholecystectomy [16]. Another example, intraoperative bleeding is an important prognostic factor in patients undergoing hepatocellular carcinoma resection for tumor recurrence and death. This is particularly important, especially with the high tendency of the liver to bleed intraoperatively due to its high vascularity and the lack of ability of sinusoidal smooth muscle contraction, and thus the inability to achieve good vasoconstriction after vascular injuries [17]. Several studies have investigated the role of SURGICEL® in hemostasis during hepatobiliary surgeries. For example, a study by Masci et al. evaluated the role of ORC gauze in achieving hemostasis during laparoscopic cholecystectomy. The study demonstrated that the use of ORC helped control bleeding effectively in all patients within minutes, even in those who had comorbidities or used oral anticoagulants. This, in turn, helped reduce the need for conversion from laparoscopic to open surgery and certainly reduced the postoperative morbidity and mortality [16].

Another study by Genyk et al. evaluated the role of SURGICEL® in the secondary treatment of local bleeding in patients undergoing hepatic resection. Although the fibrin sealant patch was shown to be superior to SURGICEL® in terms of hemostasis and bleeding control in the study, SURGICEL® was shown to be able to achieve hemostasis in 76.4% of adult patients in 5 minutes. This proportion rose to approximately 90% after 10 minutes. The results were also similar in pediatric patients, with 77.8% of patients achieving hemostasis within 5 minutes and 89% of patients within 10 minutes. This study also demonstrated that SURGICEL® was well tolerated by both groups, and no serious side effects were reported to be associated with its use [17].

Besides the use of SURGICEL® in operative hepatic procedures, it could be used in the management of bleeding control after hepatic injuries. In a study by Khoshmohabat et al., SURGICEL® was used for the management of uncontrolled hemorrhage after grade 4 lacerations in Wistar rats. This study showed that, when compared to simple gauze, SURGICEL® increased the formation of fibrotic liver tissue at the laceration site, and thus contributed to lowering the possibility of recurrent bleeding. This type of fibrotic response induced by SURGICEL® holds promise for improving hepatic hemostasis. As we advance toward personalized medicine, the integration of such adjunctive tools into clinical practice may enhance patient outcomes in hepatic surgery and hepatic trauma management [18].

SURGICEL® has also proved useful in achieving hemostasis in cases of splenorrhaphy following low-grade splenic injury, along with other topical hemostatic materials [19]. It was also shown to control bleeding in patients with iatrogenic splenic injuries during laparoscopic urological surgery without postoperative complications [20].

Clinical outcomes in urological procedures: Homeostatic agents play an important role in many urological surgeries. They have different indications, and many studies have evaluated their uses, effectiveness, and adverse events [21]. In a study by Johnston et al., the authors performed a comparison between different hemostatic agents after partial nephrectomy in a hypertensive porcine model. This study demonstrated that the sutured bolster of SURGICEL® was effective in achieving hemostasis after small and medium resections. Additionally, only the use of FloSeal followed by a sutured bolster of SURGICEL® technique was shown to be effective in achieving hemostasis after large resections [22]. Another study by Finley et al. evaluated the effectiveness of the Tisseel-Surgicel sandwich in achieving sutureless hemostasis during laparoscopic wedge resection (LWR) for small renal masses. The study showed that this approach helped in achieving excellent hemostasis in all patients who underwent LWR [23].

SURGICEL® was also shown to be effective in achieving hemostasis and reducing bleeding in cases of suprapubic simple open prostatectomy. A study by Kazemi et al. evaluated the role of SURGICEL® in achieving hemostasis after transvesical prostatectomy. The authors suggested that the postoperative blood loss in bladder lavage fluid was significantly lower in the SURGICEL® group (72.56 ± 32.53 g) compared to the control group (120.83 ± 46.66 g). This reduction in blood loss was not associated with increasing the risk of postoperative complications [15]. Interestingly, SURGICEL® can even be used to stop bleeding following newborn circumcision if pressure alone is inadequate [24].

A recent study by Wolfe et al. discussed the role of SURGICEL® in penile surgeries. The study investigated its efficacy in reducing postoperative corporal bleeding following inflatable penile prosthesis surgery. Scrotal hematoma is one of the most common complications following penile prosthesis surgery, and it usually presents as scrotal pain and discomfort in the early postoperative period. SURGICEL® use was shown to decrease the incidence of scrotal pain and swelling postoperatively with no significant increase in the risk of postoperative complications in comparison to the non-ORC group, along with a significant decrease in the output of scrotal drain [25].

Clinical outcomes in colorectal procedures: Effective hemostasis is also important in colorectal surgeries. This is particularly important in surgeries and procedures that can lead to substantial mucosal defects, such as endoscopic submucosal dissection (ESD), and can result in increased risk of complications, including delayed bleeding and perforation. A study by Myung et al. evaluated the role of SURGICEL® in reducing complications after colorectal ESD. SURGICEL® was shown to be effective in reducing some complications and mean hospitalization periods. In this study, SURGICEL® use led to a decrease in the risk of many postoperative complications, such as delayed bleeding, postoperative fever, and C-reactive protein level. It also reduced the duration of hospital stay, enhanced patient safety, and contributed to better clinical outcomes [26].

SURGICEL® is mainly used as a hemostatic material. However, several studies have been conducted to investigate the different properties and the possible uses of SURGICEL®. In a study by Larsson et al., SURGICEL® was shown to reduce the incidence of postoperative intraperitoneal adhesions in rats. Despite that, SURGICEL® was not approved to be used for this purpose clinically [27].

Specialized complications in obstetrics and gynecology: ORC is a well-recognized tool useful for hemostasis in various surgical procedures in obstetrics and gynecology. Several studies have reported how SURGICEL® was used to achieve hemostasis in various gynecological surgeries such as hysterectomies, ovarian cystectomies, myomectomies, and incontinence procedures. In a study by Sharma et al., the authors investigated the role of SURGICEL® in the management and control of bleeding after small uterine perforations. This study showed that SURGICEL® succeeded in controlling bleeding in all women with fundal, anterior, posterior, and upper lateral uterine wall perforations within approximately two minutes. Due to extensive blood loss in patients with low lateral uterine perforations, hemostasis was difficult to achieve using SURGICEL® alone. Thus, the authors used polyglactin 910 sutures on the bleeding points to achieve good hemostasis. All patients were discharged 24 hours later in good condition, and no complications associated with SURGICEL® use [28].

A case series by Awonugaet al. discussed the use of SURGICEL®/Penrose pack in four patients with uncontrollable hemorrhage after gynecological procedures. One of them occurred after cesarean hysterectomy, and the rest after debulking surgery for advanced gynecologic malignancies. All of them underwent uterine or hypogastric artery ligation to control bleeding with no success. This packing technique helped in the control of bleeding in all patients and worked as a continuous bleeding monitor postoperatively. The packing materials were removed 48 hours postoperatively in three patients at the bedside with no acute complications. In the last patient, the pack was removed in the operating room 96 hours post-surgery due to fear of left ureteric injury. All patients were doing well postoperatively and reported no complications [29].

In addition to these studies, several case reports in the literature have reported different uses for SURGICEL® in various surgical procedures. Sharma et al. reported the use of SURGICEL® to control intraoperative bleeding in patients who underwent laparoscopic endometriotic cystectomy with good results [30]. Another case report discussed the use of SURGICEL® to achieve hemostasis in patients with constant oozing of blood from the uterine cesarean section incision. SURGICEL® successfully helped in bleeding control despite failure of other hemostasis-achieving techniques such as oxytocin drip, prostaglandin F2, alpha, and others.

Neurosurgery

Hemostasis plays a critical role throughout neurosurgery. In microneurosurgery, maintaining a bloodless field under an operating microscope facilitates fast and effective surgery, significantly reducing the risk of postoperative hemorrhage. In spine surgery, while bleeding is an inevitable part of every procedure, uncontrolled bleeding can lead to severe consequences, including potential spinal cord compression and damage to the central nervous system [31]. Additionally, applying hemostatic material to the neurosurgical bed is crucial to prevent hematoma formation due to wound bleeding [32]. Traditionally, hemostasis was achieved through electrical stimulation, which, although effective, could cause vessels to retract into the brain tissue, complicating coagulation or even causing brain tissue to liquefy, resulting in irreparable damage [33]. Although mechanical methods of maintaining hemostasis are still used, such as patient positioning, bone plugging, suction, and metal clips, neurological hemostasis and hemorrhage control are mostly achieved through bipolar coagulation, allowing for precise coagulation of small vessels and safe use in patients with pacemakers and defibrillators. Within neurosurgery, SURGICEL® is commonly used to control capillary, venous, or smaller arterial bleeding as it acts as a matrix to facilitate clot formation [12]. It is commonly used in spinal procedures and is preferred to bipolar cautery in intraspinal surgeries, as occlusion of the vessel lumen or minimal bleeding within the spinal canal may lead to catastrophic neurological damage. In translabyrinthine and transcochlear approaches to the posterior cranial fossa, medium tears of the sigmoid sinus can be managed by placing SURGICEL® over the opening [34]. Intracranial hemorrhage surgery is another function of SURGICEL® as it aids in hemostatic effect [32]. Additionally, SURGICEL® is typically preferred in the management of pineal region tumors. In these circumstances, careful placement should be performed to avoid floating of the strip and obstruction of the cerebral aqueduct upon filling of the third ventricle with cerebrospinal fluid [35]. SURGICEL® is compatible with minimally invasive surgery as the fabric material allows easy passage through the laparoscopic trocars to the desired tissue.

Compared to other hemostatic agents, Surgicel™ offers an exceptionally high level of tissue compatibility in neurosurgical applications, with blood absorption beginning within a day after application, and full absorption occurring within four to eight weeks [32,36]. However, SURGICEL® use is not without risks. Notably, there is a risk of granuloma formation, particularly during intracranial hemorrhage debridement surgery, which may lead to the development of tumor-like, space-occupying lesions within the surgical bed [32]. Granulomatous reactions to oxidized cellulose have been reported 2 months and 12 months after the removal of intracranial meningiomas. Both cases presented with contrast-enhancing, space-occupying lesions on CT scans, initially suspected as tumor recurrence [37]. The appearance of retained oxidized cellulose on CT scans can mimic that of an abscess or tumor, increasing the risk of erroneous diagnosis. Numerous studies have highlighted the misdiagnosis of SURGICEL®-related granulomas as brain tumors, particularly where granuloma formation occurs after cerebral tumor resection. Additionally, in spinal surgery contexts, when oxidized cellulose is used near nerve structures in confined bony areas, such as foramina, spinal cord, or optic chiasm, there is a potential risk of nerve damage [38]. SURGICEL®’s effectiveness may be limited under certain conditions, such as in patients on anticoagulant therapy or those with intraparenchymal hemorrhages [39,40].

Orthopedic Surgery

SURGICEL® is commonly used in orthopedic surgery to control bleeding and promote hemostasis, particularly in joint replacement surgeries, spinal surgeries, and trauma surgeries in which patients are more vulnerable to substantial blood loss. Despite that, many studies in the literature have discussed the role of SURGICEL® in other orthopedic surgeries, such as upper extremity surgeries and others. In addition to its role in hemostasis, SURGICEL® was used for many other purposes in orthopedics [41]. In a study by Degreef et al., the authors discussed the use of SURGICEL® as an adhesion barrier in patients with Dupuytren contracture. All patients in the study underwent surgical fasciectomy to relieve the Dupuytren contracture. In about half of them, the authors used the SURGICEL® as an adhesion barrier with good results and significant improvement in postoperative range of motion immediately after surgery and on follow-up one year later [42].

SURGICEL® was also shown to aid in osseous regeneration and bone healing in several animal studies. In a study by Dias et al., the authors examined the role of SURGICEL® in bone regeneration in sheep. The study concluded that SURGICEL® was effective in the healing of bone defects created in the tibia and femur of the sheep within six to eight weeks [43]. Another study by Nooh et al. assessed the effects of SURGICEL® on bone healing in goats’ feet in comparison to bone wax. They suggested that SURGICEL® had greater efficacy over bone wax in terms of induction of granulation tissue with new bone formation and thus osseous healing [44]. Based on these studies, Abdelkhalek et al. conducted a study to evaluate the effectiveness and role of SURGICEL® in the management of segmental skeletal defects in high-energy trauma patients. Segmental skeletal defects are not easy to treat. They usually require long surgeries and multiple-stage reconstruction surgeries, and are associated with multiple complications. In this study, the authors aimed to assess the results of bone graft in SURGICEL® as a synthetic membrane for the reconstruction of segmental skeletal defects in one-stage surgery. The study showed that, except for three patients, all defects were completely healed after one surgery within 6-13 months, with an average of eight months. The majority of patients regained a good range of motion after physiotherapy. SURGICEL® helped achieve good bone healing within post-traumatic segmental skeletal defects and reduced the number of surgeries required to manage those patients effectively [45].

Oral and Maxillofacial Surgery

**Figure 2:**
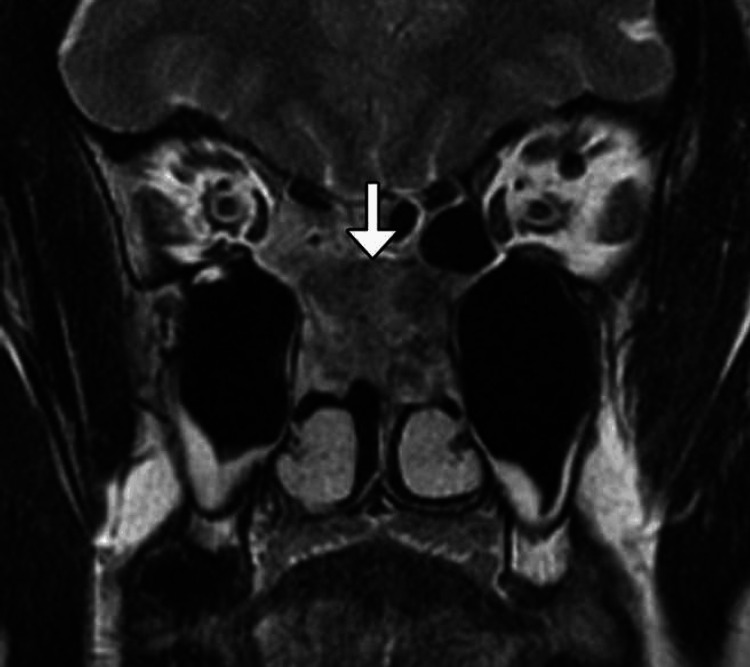
SURGICEL® nasal packing. This image illustrates the use of SURGICEL® as nasal packing to control bleeding during procedures involving the nasal cavity, demonstrating its application in managing epistaxis and ensuring hemostasis. This is an original figure from the authors.

Hemostasis is crucial for ensuring safety in all surgical practices, particularly in oral and maxillofacial surgery, where procedures involve the head and neck, areas known for their extensive vascular supply and the critical need to maintain a clear airway. SURGICEL® is broadly employed in oral and maxillofacial surgery to limit arterial bleeding within bony structures, particularly from the inferior alveolar artery. The loss of hemostasis following dentoalveolar procedures can significantly compromise the airway and lead to hypovolemia, even in patients without known bleeding disorders [46]. Orthognathic surgery poses a higher risk of substantial blood loss, which tends to increase with the complexity and number of surgical sites [47]. Therefore, the utilization of effective hemostatic agents is essential. The selection of a hemostatic agent is typically based on the severity of the bleeding encountered during surgery and any pre-existing bleeding disorders in the patient’s history. While SURGICEL® is infrequently used in oral surgery, it is sometimes necessary when bleeding cannot be managed by conventional packing and suturing alone. SURGICEL® is occasionally used during tooth extraction procedures due to its efficacy in sealing wounds. However, its use has been associated with a higher incidence of dry socket. Although small amounts of SURGICEL® are absorbed within about eight weeks, larger quantities may take longer to resolve. Additionally, its acidic pH can delay resorption, potentially leading to post-surgical adhesions and other complications [48]. In addition to tooth extraction procedures, SURGICEL® can be used to control postoperative bleeding in patients undergoing tonsillectomies and salivary gland surgeries, as it has been shown to reduce the incidence of bleeding in the head and neck operative sites [49,50]. A study by Cetiner et al. evaluated the efficacy of SURGICEL® in reducing postoperative bleeding and pain after tonsillectomies, as bleeding and pain are common complaints among these patients. The study concluded that SURGICEL® significantly decreased the incidence of postoperative bleeding. However, the authors noted an increase in postoperative pain during the first few days after surgery. This increase was attributed to the use of suturing in the ORC group, which, in turn, caused more inflammation and pressure at the wound site [49]. Lastly, SURGICEL® could be used in the surgical treatment of epistaxis. SURGICEL® can be placed over the cauterization site to promote hemostasis (Figure 2) [51]. While effective in these varied roles, SURGICEL®’s performance relative to newer biological sealants and synthetic alternatives warrants a direct comparison.

Comparison with other hemostatic agents

Hemostatic materials encompass a variety of forms, including fibrin glue, polysaccharide, and oxidized cellulose [52]. These materials are categorized into patch type, particle powder type, fluid sealing type, and tactile expansion type, designed to rapidly achieve hemostasis without causing allergic reactions or toxicity. Their morphological characteristics, such as sponges for tamponade and powders for fine granular coverage, determine their suitability for specific wound types and bleeding situations [52].

Comparatively, SURGICEL® has been scrutinized against other hemostatic agents such as Vivostat® and Fibrin Sealant Grifols, revealing varying degrees of effectiveness and reliability. Vivostat® demonstrated superior hemostasis efficacy and reliability over SURGICEL®, with significantly shorter mean times to hemostasis and higher patient success rates without needing additional hemostatic measures [53]. Vivostat® showed a mean time to hemostasis significantly shorter than SURGICEL® (1.6 vs. 3.3 minutes, p < 0.0001), indicating faster and more reliable hemostasis [53]. Similarly, Fibrin Sealant Grifols outperformed SURGICEL® in achieving hemostasis during hepatic resections, with higher rates of hemostasis within shorter timeframes and no significant difference in adverse events between the two [54]. These comparisons underscore the importance of selecting the appropriate hemostatic agent based on surgical needs and outcomes.

Comparing SURGICEL® to microfibrillar collagen powder, the latter demonstrated superior efficacy in reducing intraoperative blood loss and postoperative drainage volumes in a cardiac surgery context. Specifically, patients treated with microfibrillar collagen powder (Colgel) experienced significantly less chest tube drainage in the first 24 hours (373 ± 143 mL) compared to the SURGICEL® group (571 ± 144 mL). Additionally, the Colgel group required markedly fewer packed red blood cell units (28 units) than the SURGICEL® group (120 units), especially notable in the initial three postoperative hours [55]. These findings highlight microfibrillar collagen powder's benefits, including easy application, cost-effectiveness, and significant reduction in blood loss, demonstrating yet another superior alternative to SURGICEL®.

In comparing non-regenerated and regenerated (SURGICEL®) oxidized cellulose hemostatic agents, a study found that non-regenerated oxidized cellulose exhibits superior hemostatic performance. This superiority was evident in both non-heparinized and heparinized conditions, with notable differences in the time to achieve hemostasis. Non-regenerated oxidized cellulose not only required less time to halt bleeding but also demonstrated greater hemostatic success early after application. Given its equivalent bactericidal effects and enhanced hemostatic properties, non-regenerated oxidized cellulose may become the preferred choice in surgical applications after further clinical validation [56].

Metabolism, clearance, and radiographic appearance

In general, the SURGICEL® mass is composed of two major components, soluble polyan-hydroglucuronic acid, the functional unit, and fibrous component [57]. The acidic part is typically lost within the first day after implantation by systemic clearance. After a week, multinucleated giant cells (macrophages) invade the SURGICEL® mass and facilitate clearance of the fibrous residues via phagocytosis [37]. It is walled-off by granulation tissue at 14 days, and further surrounded by fibroblastic tissue at 21 days [58]. The mass is totally reabsorbed in four to eight weeks postoperatively. Absorbability is highly dependent on the quantity of the material used and the regional blood flow to the tissue bed. As a result, immediate postoperative imaging studies of patients in which SURGICEL® has been used may still reveal the presence of hemostatic material.

The literature on the radiologic appearance of SURGICEL® remains scant. This is thought to be due to the rapid biodegradation of SURGICEL®, hence limited identification, and the wide variety of surgeries and anatomical locations in which they are utilized [10]. It should be noted that air can be trapped during the degradation process, resulting in multiple radiological patterns that resemble those of an abscess. In this section, we summarize the radiographic features.

Plain Radiography (X-ray)

On a conventional radiograph, SURGICEL® can be seen as a radiopaque area (linear band of increased density), particularly when large quantities are used [59]. More importantly, air can be trapped within the SURGICEL® fabric, admixed with the absorbed blood at the site of implantation, producing a gas-containing hematoma. The appearance of such hematomas, notably in the abdominal cavity, raises concern about the possibility of an abscess caused by gas-forming bacteria, bowel perforation, and residual gas after removal of a drain [60].

Ultrasonography

SURGICEL® may appear as a heterogeneous hypodense echogenic mass with posterior reverberation artifact on ultrasound and can be mistakenly identified as a gas-containing abscess in the postoperative setting. In addition, the mass typically possesses poorly defined margins, and surrounding free fluid may be present, reflecting saturation of the SURGICEL® fabric with blood. Of note, the trapped gas bubbles within the folds of SURGICEL® can coalesce and create large, bright reflective surfaces. Short-path reverberation artifact between the trapped gas bubbles is responsible for the acoustic noise that can be visualized posterior to the mass. SURGICEL® is frequently used in liver transplant procedures. Subsequently, the sonographic appearance of SURGICEL® is of particular importance after liver transplant, as these patients require early and frequent postoperative sonographic evaluations to assess graft blood flow [61,62].

In cases of SURGICEL®-induced granulomas, the resultant mass typically has fewer sonographic features as described above; however, it can still reveal echogenicity despite the absence of trapped air. This suggests that the echogenicity may be attributed to the oxidized cellulose itself. Echogenic heterogeneity is typically appreciated and is due to disproportionate reabsorption rate in different portions of the fabric, where parts with more blood saturation show a faster dissolution rate [61].

Computed Tomography

In the early postoperative period, SURGICEL® may also mimic an abscess on CT, potentially resulting in futile attempts at drainage [62]. Unlike abscesses, SURGICEL® mass lacks an enhancing margin. A well-demarcated, focal “collection” containing a mixture of centrally located gas foci and soft tissue with mixed attenuation can be seen in or adjacent to the operative site. Surrounding free fluid may also be present [63]. On the other hand, the new, non-regenerated form of SURGICEL® has a more homogenous appearance compared with SURGICEL® Original in non-contrast CT imaging. Although it is challenging to differentiate SURGICEL® from abscesses on CT imaging, the absence of fluid collection or air-fluid level is characteristic of SURGICEL®. Other features that assist in differentiation of SURGICEL® from an abscess include an atypical pattern of gas collection (often linear or punctate due to air trapping vs. scattered in abscesses) without an air-fluid level [64].

Granulomatous reactions can occur to the oxidized cellulose several weeks to months after placement. Intracranially, this can mimic tumor recurrence with contrast-enhancing, space-occupying lesions on CT scan, putting patients at risk for unnecessary surgery [37]. In thyroid resection procedures, there have been cases where this granulomatous reaction mimics thyroid tumor post-thyroidectomy, requiring fine-needle aspiration to diagnostically evaluate the granuloma [65]. Therefore, tumor-like SURGICEL® reaction should always be taken into account when evaluating postoperative imaging studies following surgeries for intracranial hemorrhaging [32]. In the abdominal cavity, SURGICEL® material can become infected with coliform bacteria or gas-forming organisms. Radiologic features characteristic of an abscess, such as air-fluid level, fat stranding, and rim-enhancing fluid collection, can be appreciated on CT at the operation site. These findings should be investigated in a clinical context. Systemic symptoms such as fever and local tenderness should further raise the suspicion of infection (abscess), and spontaneous drainage combined with appropriate antibiotic therapy may be required.

Magnetic Resonance Imaging

Signal intensity of SURGICEL® compared to neighboring structures differs in T1- and T2-weighted images. SURGICEL® has a short relaxation time on T2-weighted images, resulting in low signal intensity in the early postoperative period and is therefore demonstrated as a local area of hypointensity [62,66,67]. However, it is typically hyperintense compared to muscles on T2-weighted images [67]. On T1-weighted images, SURGICEL® appears hypointense compared to adjacent tissues (Table 1). Over time, the homogeneity of the hemostat increases as the size decreases, though signal intensity remains stable. Occasionally, SURGICEL® may invoke foreign-body granulomatous reactions months after surgery, resulting in an enhancing mass at the site of placement [68].

**Table 1:** MRI appearance of SURGICEL® compared to different surrounding tissues.

	T1-weighted images	T2-weighted images
Hyperintense	-	Liver, muscles
Hypointense	Most tissues (e.g., fat, muscles, liver, kidneys)	Adipose tissue, kidneys

If SURGICEL® is used in lumbar laminectomies, particularly when placed against the dura, remarkable spinal canal compression is considered a normal finding unless associated with clinical symptomatology. In such circumstances, the dural compression produced by the retained SURGICEL® may be greater than that evident in the preoperative period [69]. This should be differentiated from epidural hematomas, which are typically identified as hyperintensity on T2-weighted MRI. In addition, subdural hematomas are more widely distributed around the cord compared to SURGICEL® mass, which classically appears as a focal mass compressing the cord. Epidural hematomas may sometimes be managed conservatively, whereas masses of SURGICEL® must always be removed to salvage spinal cord function [66]. Recognizing these unique imaging signatures is the critical first step in establishing an accurate differential diagnosis and preventing postoperative mismanagement.

Differential Diagnosis

Before establishing a differential diagnosis, an appropriate history should be sought by the operating surgeon regarding whether SURGICEL® hemostatic material has been used. This information should be relayed to the interpreting radiologist to facilitate diagnosis and guide clinical management. Although the majority of case reports in the literature on postoperative radiological appearance of SURGICEL® demonstrated misinterpretation as post-surgical abscesses or local tumor recurrence, potential etiologies, such as other retained surgical hemostatic materials, hematomas, and ongoing oozing, should always be taken into account [64,65].

Interestingly, accidentally retained non-absorbable surgical sponges can share similar sonographic features with SURGICEL® postoperatively. Ultrasonography of these sponges would reveal an echogenic mass with well-demarcated posterior acoustic shadowing [61]. However, conventional radiography, ultrasonography, and CT scan provide limited ability to reliably distinguish between SURGICEL® and other similar pathological processes that may necessitate urgent intervention, particularly abscesses. Alternatively, MRI may help differentiate SURGICEL® from an abscess and therefore avoid unnecessary attempts at aspiration. Clinical symptoms such as fever, chills, local pain or tenderness, anorexia, and fatigue are typical for deep abscesses. In these cases, subsequent percutaneous aspiration may be necessary. Furthermore, the SURGICEL® material can be infected as it does not typically have antiseptic properties, and therefore may lead to abscess formation.

Complications

Although the utilization of SURGICEL® in surgical procedures is generally considered safe and effective, it is associated with potential complications that necessitate careful consideration. These complications can vary depending on the specific surgical context and patient characteristics. Surgeons should be mindful of the placement and quantity of SURGICEL® used, especially in high-risk patients or procedures. Postoperative monitoring is essential for the early detection and management of complications [70].

A recent systematic review was conducted by Masoudi et al. to evaluate the safety and potential complications associated with SURGICEL® use in various surgical procedures. After analyzing the data from 7,242 participants from multiple case reports, retrospective, prospective studies, and randomized control studies, the authors reported that SURGICEL® use was associated with various complications. The average time for follow-up in these studies was about two years [70].

The most common adverse event reported in patients after SURGICEL® use was SURGICEL® -induced masses (Surgicelomas). In vivo, SURGICEL® is gradually absorbed by the body after surgery within 10-14 days with some variation due to differences in patients’ characteristics and procedures, in addition to differences in the amount of SURGICEL® used. In fact, some studies reported the presence of SURGICEL® on imaging more than a month postoperatively. This suggests that the body’s response to SURGICEL® still needs extensive studies and investigations [71]. Incomplete absorption can lead to a foreign body reaction, potentially resulting in granuloma, hematomas, cysts, or abscess formation at the application site. SURGICEL® induced masses were misdiagnosed as tumors in many patients, and some of them underwent unnecessary surgical evaluation for these masses, especially in cases where the treating physician was unaware of SURGICEL® use in a recent surgical procedure [70].

Hemorrhagic complications were also reported in many studies. This is thought to be related to inadequate hemostasis with SURGICEL® use, which can result in hematoma formation at the surgical site, increasing the risk of infection and other complications. In a study by Peyronnet et al., the authors showed that the use of SURGICEL® and other hemostatic agents did not prevent the incidence of hemorrhage in patients who underwent robotic partial nephrectomy [72].

Several studies have reported pain, cardiovascular, nervous system, and hepatobiliary complications after SURGICEL® use. This is thought to be associated with compression of surrounding structures in surgeries where SURGICEL® is in proximity to nerves or vital organs, which can cause nerve damage, vascular compromise, or other significant complications [70]. In a study by Arora et al., the authors reported that two patients had cardiac arrest after SURGICEL® use due to compression of the heart by the swollen SURGICEL® mass after cardiovascular surgery [73]. Additionally, several studies reported various neurological symptoms due to compression of nearby neural structures. Cauda equina syndrome and delayed paraplegia were reported in many patients after spinal and thoracic surgeries [74,75].

Despite the fact that SURGICEL® has antibacterial properties, infection was also among the most common complications reported after SURGICEL® use. It can act as a nidus for infection, particularly in surgeries with contamination risk, facilitating bacterial adherence and proliferation, which can manifest as local infection at the surgical site [45,70]. Other less common complications found to be associated with SURGICEL® include chorioamnionitis, urinary leaking, fistulas, erectile dysfunction, and renal failure [70]. Allergies, though uncommon, have been shown in literature and should be thoroughly evaluated [70,76].

Limitations

This narrative review has several limitations. First, some referenced studies are more than 10 years old; however, this reflects the limited and relatively static body of literature surrounding SURGICEL®, a product that has been in clinical use for decades. As a result, earlier foundational studies remain relevant and continue to inform current surgical practice. Second, as a narrative review, this article does not follow a systematic search strategy or quantitative synthesis, which may introduce selection bias and limit reproducibility. Finally, variability in study designs and outcome measures across the included literature may restrict direct comparisons and the generalizability of findings. Future research should prioritize prospective, standardized, and comparative studies evaluating SURGICEL® against newer hemostatic agents, with particular emphasis on long-term outcomes and radiologic differentiation to reduce postoperative misdiagnosis.

## Conclusions

In summary, SURGICEL® is a widely used local hemostatic agent in nearly all surgical specialties, including neurosurgery, thoracic surgery, and orthopedics. Proper communication between the operating surgeon and the interpreting radiologist is crucial when SURGICEL® is used. MRI is a reliable tool to discern the presence of SURGICEL® versus an abscess. Therefore, knowledge of imaging characteristics as well as a patient’s clinical symptoms is essential to prevent additional costs and risks to the patient associated with unnecessary workup and/or surgical interventions. Future clinical research should focus on comparative trials to establish standardized imaging protocols for bioabsorbable hemostats, which will further minimize diagnostic ambiguity and optimize patient safety.
